# Genome-Wide Association Study Dissecting the Genetic Architecture Underlying the Branch Angle Trait in Rapeseed (*Brassica napus* L.)

**DOI:** 10.1038/srep33673

**Published:** 2016-09-20

**Authors:** Chengming Sun, Benqi Wang, Xiaohua Wang, Kaining Hu, Kaidi Li, Zhanyu Li, San Li, Lei Yan, Chunyun Guan, Jiefu Zhang, Zhenqian Zhang, Song Chen, Jing Wen, Jinxing Tu, Jinxiong Shen, Tingdong Fu, Bin Yi

**Affiliations:** 1 Huazhong Agricultural University, National Key Laboratory of Crop Genetic Improvement, National Sub-center of Rapeseed Improvement in Wuhan, Wuhan, 430070, China; 2 Hunan Agricultural University, College of Agronomy, Changsha, 410128, China; 3 Jiangsu Academy of Agricultural Science, Key Laboratory of Cotton and Rapeseed, Nanjing, 210014, China

## Abstract

The rapeseed branch angle is an important morphological trait because an adequate branch angle enables more efficient light capture under high planting densities. Here, we report that the average angle of the five top branches provides a reliable representation of the average angle of all branches. Statistical analyses revealed a significantly positive correlation between the branch angle and multiple plant-type and yield-related traits. The 60 K *Brassica* Infinium^®^ single nucleotide polymorphism (SNP) array was utilized to genotype an association panel with 520 diverse accessions. A genome-wide association study was performed to determine the genetic architecture of branch angle, and 56 loci were identified as being significantly associated with the branch angle trait via three models, including a robust, novel, nonparametric Anderson-Darling (A-D) test. Moreover, these loci explained 51.1% of the phenotypic variation when a simple additive model was applied. Within the linkage disequilibrium (LD) decay ranges of 53 loci, we observed plausible candidates orthologous to documented *Arabidopsis* genes, such as *LAZY1*, *SGR2*, *SGR4*, *SGR8*, *SGR9*, *PIN3*, *PIN7*, *CRK5*, *TIR1*, and *APD7*. These results provide insight into the genetic basis of the branch angle trait in rapeseed and might facilitate marker-based breeding for improvements in plant architecture.

Rapeseed (*Brassica napus* L., 2n = 38, genome AACC) is one of the most important oilseed crops worldwide. Branch angle, or the adaxial angle of the branch to the stem, is a key morphological trait that shapes the canopy architecture and thus influences yield. This notion is best exemplified through the continuous increase in grain yield of US corn over the past century, which reflects the adaptation of hybrid upper leaf angles that continually increase plant densities^1^. Plants with adequately small branch angles display compact canopy architectures, and these plants are therefore more suitable for high-density planting. Furthermore, at later growth stages, siliques replace leaves as the main photosynthesis organ^2^, so a compact canopy architecture maintains light capture under high densities by minimizing shading by adjacent plants. Accordingly, clarifying the genetics determining branch angles will be of great value for continuous improvements in rapeseed architecture.

Branch angles are primarily regulated through shoot gravitropism, which is also referred to as the gravitropic set-point angle (GSA)^3^ and includes four phases: gravity perception, gravity signal transduction, auxin asymmetric redistribution, and organ curvature^4^. To date, numerous genes that influence branch (tiller) angle have been identified in model plants. For example, *Arabidopsis* genes *sgr1-sgr9* cause attenuation of the shoot gravity perception capacity and alter branch angles^5^. *LAZY1* has been implicated in the regulation of the rice tiller angle, and loss of *LAZY1* function alters the distribution of endogenous IAA in the shoot, which reduces shoot gravitropism and leads to a tiller-spreading phenotype^6^. A recent study has also identified a role for the *Arabidopsis LAZY1* orthologue in branch angle regulation^7^. *TAC1*, another branch (tiller) angle gene that was first identified in rice^8^, is derived from the same *IGT* gene family as *LAZY1*^9^ and recent studies have suggested that orthologues of *TAC1* control branch angles in *Arabidopsis* and peach^9^. Moreover, a recent study in *Arabidopsis* showed that mutants with defects in auxin homeostasis or auxin response genes, such as *wei8 tar2*, *tir1-1*, *afb4-2 afb5-5*, and *arf10-3 arf16-2*, have altered branch angles^10^. However, despite the increasing understanding of branch angle mechanisms in model plants, the genetic basis of branch angle in rapeseed has not been elucidated, a situation that reflects the complexity of genetic studies of polyploid plants.

A genome-wide association study (GWAS) provides a methodical analysis of the genetic architecture of complex traits in crops. GWAS identifies the underlying QTLs at a relatively high resolution, taking full advantage of ancient recombination events^11^,^12^. To date, GWAS has been successfully performed in many crops, including rice^13^, maize^14^,^15^ and rapeseed^16^,^17^,^18^. Among the proposed statistical approaches for GWAS, the mixed linear model (MLM) is a popular method that can eliminate the excess of low *p*-values for most traits^19^,^20^. However, MLM can lead to false negatives by overcompensating for population structure and kinship^21^, and MLM has also limited statistical power to detect rare alleles, which in fact constitute a substantial proportion of the natural variation^22^ and have potentially large phenotypic effects^23^. Accordingly, a complementary strategy, the Anderson-Darling (A-D) test, has recently been proposed to rectify these shortcomings^24^. The A-D test is a novel nonparametric statistical method that offers a higher power than MLM for traits that have abnormal phenotypic distributions and are controlled by moderate effect loci or rare variations^24^.

In the present study, we investigated correlations between the angles of branches at different positions and between these angles and other important agronomic traits. Genome-wide SNPs of this panel were assessed using the 60K *Brassica* Infinium^®^ SNP array, and the corresponding phenotype was evaluated in four environments. GWAS was performed with 520 diverse accessions to identify underlying QTLs that contribute to rapeseed branch angle variations. A total of 56 loci that were significantly associated with the branch angle trait were identified by three association methods: MLM, the general linear model (GLM) and the A-D test. Considerable candidate genes were identified based on LD decay range of these loci, including multiple orthologues of well-characterized *Arabidopsis* genes. This study demonstrates that GWAS can be used as an effective approach for dissecting complex quantitative traits in rapeseed.

## Results

### Phenotypic variations in branch angle among accessions

High positive correlations were observed among the angles of different branches, indicating similar genetic control (Table 1). In general, the correlation coefficient between branches was reduced with decreasing physical proximity. For example, the angle of the second branch from the top showed a maximum correlation with the angle of the third branch (r = 0.72) and then with the fourth and fifth branches (r = 0.61, 0.53, respectively). In addition, we observed that the average angle of a different number of branches from the top was significantly positively correlated with the average angle of all branches, particularly when the branch number reached five (r = 0.93, Table 1). Thus, it is feasible to measure the five top branches as a representation of the branch angle phenotype. Phenotypic data for 30 individual plants are presented in Supplementary Data S1.

We collected phenotypic data for the association panel in four environments; the trial performed at the Changsha farm was the only trial with phenotypic data across two growing seasons (Table 2). Extensive phenotypic variations were observed for branch angles in the association panel, as indicated by the descriptive statistics shown in Table 2. In the four environments, the branch angle varied from 21.7 ± 1.9° to 71.7 ± 4.8°, with an average ranging from 40.3 ± 6.3° to 43.2 ± 6.3°. The coefficient of variation was constant in the different environments and ranged from 14.5% to 16.2%. The phenotypic data for all accessions in the four environments as well as BLUP values are presented in Supplementary Data S2.

The branch angles of the association panel in the four environments exhibited significantly positive correlations with each other, indicating the reliability and repeatability of these phenotypic data (Supplementary Table S1). Analysis of variance (ANOVA) revealed that the genotype, environment (year and location) and genotype × environment interaction all had significant effects on the branch angle, suggesting the crucial influence of environment on branch angle regulation (Supplementary Table S2). Based on phenotypic data for the four environments, the broad-sense heritability of the branch angle was as high as 78.5%.

We then analysed the relationship between the branch angle and other important agronomic traits (only data for 2012/2013 and 2013/2014 at Changsha were available for these traits). Notably, branch angle was significantly positively correlated with plant height, which had the highest coefficient (r = 0.25, 2012/2013 Changsha), followed by branch number (r = 0.17, 2012/2013 Changsha, Table 3). Moreover, significant positive correlations were also observed between the branch angle and four yield-related traits, including the main inflorescence pod number, pod length, seed number per pod and seed yield (2012/2013 Changsha, Table 3). Similar results were observed in the 2013/2014 Changsha phenotypic data (Supplementary Table S3). Therefore, the results indicated a close relationship between branch angle and multiple plant-type and yield-related traits.

### SNP performance, quality and in silico mapping

The Illumina *Brassica* 60K Infinium^®^ SNP array was used to genotype 530 rapeseed accessions. The raw data generated using the Illumina Infinium platform were further analysed with Genome Studio software by cluster refinement with an optimum accession Call Rate > 0.7; SNP Call Freq > 0.75; Minor Freq > 0.05; AA, BB frequency > 0.03; and GenTrain Score > 0.5. Through this analysis, 520 accessions and 33,218 polymorphic SNPs (63.7%) were retained. After excluding SNPs lacking clearly defined clusters or with multiple loci in the genome, 19,167 high-quality SNPs (36.7%) genotyped across 520 rapeseed accessions were utilized for association mapping. The genotyping scores for all polymorphic SNPs are presented in Supplementary Data S3.

### Population structure and linkage disequilibrium

The population structure of the association panel was calculated for the 19,167 SNPs using STRUCTURE, and the parameters LnP(D) and Delta K suggested that the 520 genotypes could be assigned to two groups. A probability of membership threshold of 0.60 was used, and 65 and 398 lines were assigned to Groups 1 and 2, respectively, with the remaining 57 lines classified into a mixed group (Supplementary Data S4). In addition, the lm procedure in R showed that population structure accounted for 15.8% of the phenotypic variation of branch angle. The data for population structure and kinship are presented in Supplementary Data S5.

**Figure 1:**
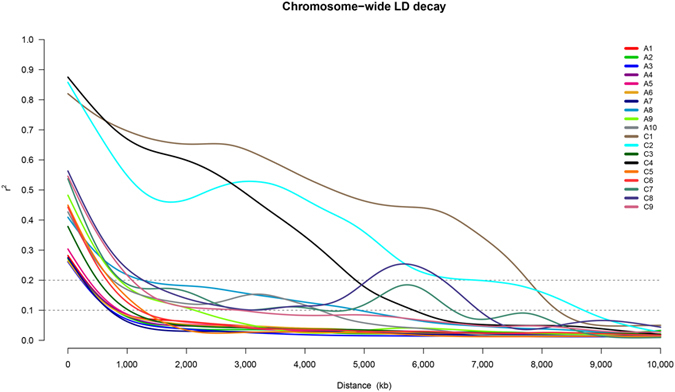
Overall chromosome-wide linkage disequilibrium (LD) decay, which is shown as a smoothed r^2^ for all marker pairs on each chromosome depending on the distance between marker pairs.

When r^2^ = 0.1, seven chromosomes (A01 to A07) exhibited comparatively modest LD, with distances ranging from 708 to 873 kb (Fig. 1, Supplementary Table S4). Chromosomes A09, C03, C05, C06 and C09 showed stronger LD, with distances ranging from 1,039 to 2,968 kb. However, particularly reinforced LD patterns were observed for chromosomes A08, A10, C01, C02, C04, C07 and C08, which presented a corresponding LD decay ranging from 4,264 to 8,704 kb. Consistent with the performance of the major A chromosomes, the A subgenome exhibited modest LD, with a distance of up to 1,046 kb, whereas the C subgenome exhibited extremely conserved LD of 7,882 kb when r^2^ = 0.1. The average LD decay for the entire genome was 6,660 kb when r^2^ = 0.1 (Supplementary Fig. S1, Supplementary Table S4).

### Genome-wide association study

**Figure 2:**
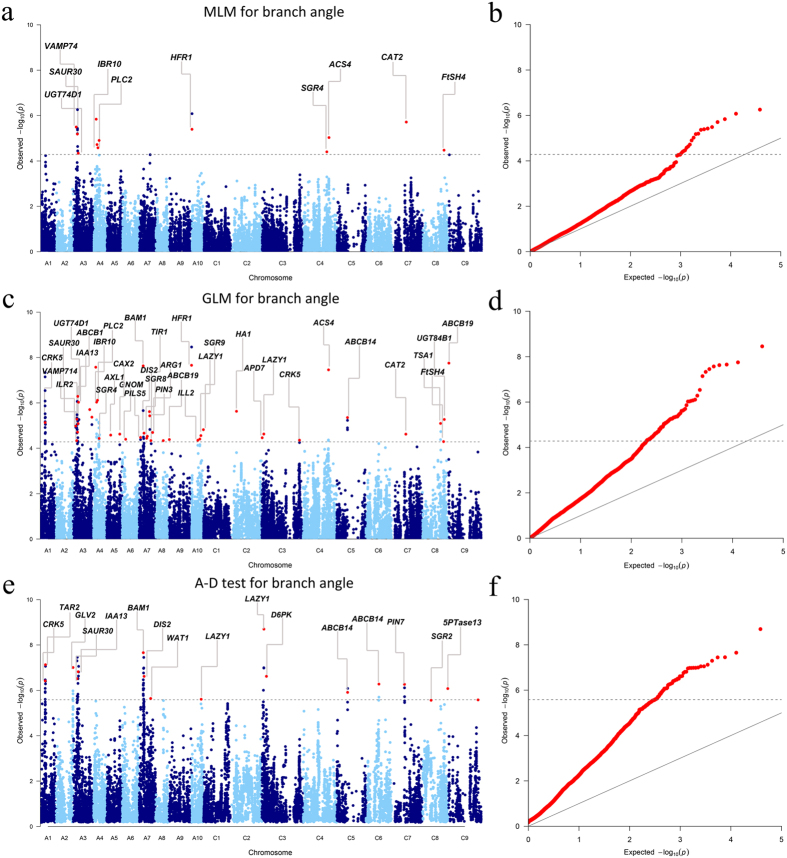
Genome-wide association study of the rapeseed branch angle. (**a**) Manhattan plot of MLM for branch angle (BLUP). (**b**) Quantile-quantile plot of MLM for branch angle (BLUP). (**c**) Manhattan plot of GLM for branch angle (BLUP). (**d**) Quantile-quantile plot of GLM for branch angle (BLUP). (**e**) Manhattan plot of the A-D test for branch angle (BLUP). (**f**) Quantile-quantile plot of the A-D test for branch angle (BLUP). The dashed horizontal line depicts the Bonferroni-adjusted significance threshold (−log_10_(*p)* = 4.3 for GLM and MLM; −log_10_(*p)* = 5.6 for the A-D test). GWAS loci are indicated with red dots, and the corresponding genes are annotated. If more than one candidate gene was identified within the observed LD decay of one locus, the most likely candidate gene is annotated in the Manhattan plot. Full gene information is provided in Supplementary Data S7.

When we initially performed GWAS with MLM using BLUP values across four environments, 19 SNPs significantly associated with branch angle were identified at a threshold of *p* < 5.2 × 10−5 (1/19,167, −log10(*p*) = 4.3); these sites correspond to 12 loci located on chromosomes A03, A04, A09, C04, C07 and C08 (Table 4, Fig. 2). Using the phenotypic data for the individual environments, MLM identified 4 additional loci on chromosomes A07, A10, C05 and C09 under the same threshold as indicated above (Table 4). Altogether, these 16 loci explained 40.3% of the total phenotypic variance.

To better utilize the genotyping and phenotyping information obtained in the present study, two permissive models, GLM and the A-D test, were introduced into our association analysis. Briefly, the two models detected 48 (GLM) and 24 (A-D test) loci significantly associated with BLUP and individual environmental data at the corresponding Bonferroni threshold, values of −log_10_(*p)* = 4.3 for GLM and −log_10_(*p)* = 5.6 for the A-D test (Table 4, Fig. 2). We then compared the consistency of the loci identified among the three methods. All loci detected using MLM were repeatedly detected through either GLM or the A-D test, and four loci were consistently detected across all three models, including Bn-A03-p6228570 on A03, Bn-A04-p4410144 on A04, Bn-A07-p13172047 on A07 and Bn-scaff_16062_1-p345501 on C05 (Table 4). A total of 16 associated loci were consistently detected between GLM and the A-D test, whereas 20 and 8 loci were exclusive to GLM and the A-D test, respectively (Table 4). Altogether, the three methods identified 56 unique loci significantly associated with the branch angle trait. Except for C01, these loci are unevenly distributed over all chromosomes. A03 and A07 both have a maximum of 10 loci, A04 and C08 five loci, and A10 and C03 three loci. The remaining chromosomes have either one or two loci (Table 4). Approximately two-thirds of the loci (38/56) are distributed in the A subgenome; the remaining loci are distributed in the C subgenome. When using a simple additive model, the 56 loci explained up to 51.1% of the phenotypic variation.

### Candidate gene mining

When using the whole genome genes as reference, two categories of genes, genes with auxin efflux transmembrane transporter activity (GO:0010329) and genes with auxin transmembrane transporter activity (GO:0080161), were found to enrich in the LD decay ranges of significant loci (false discovery rate < 0.05, Supplementary Fig. S3, Supplementary Data S6). Based on the GO annotation, *Arabidopsis* orthologue information and published gravistimulation microarray data, we further predicted candidate causal genes for loci significantly associated with the branch angle trait within the observed LD decay (r^2^ > 0.1), with 77 plausible candidate genes predicted for 53 loci. Due to the low rate of LD decay (776.8 kb on average when r^2^ = 0.1), more than one-third of the GWAS loci (20/53) have at least two candidate genes (Supplementary Data S7). For example, three candidate genes, *BnaA01g12950*, *BnaA01g13320* and *BnaA01g13580*, which are orthologous to *Arabidopsis CRK5*^25^, *ARF9*^26^ and *TRH1*^27^, were collectively identified within the LD decay of the GWAS locus Bn-A01-p7430311 (r^2^ > 0.2, Supplementary Data S7). Briefly, 48 (62.3%) candidate genes are related to auxin asymmetric redistribution, and 10 (13.0%), 5 (6.5%), and 5 (6.5%) candidate genes are involved in gravity perception, gravity signal transduction and organ curvature, respectively (Supplementary Data S7). The remaining nine genes (11.7%) are associated with ROS, phototropism, ethylene, and strigolactone (Supplementary Data S7).

In Arabidopsis, *sgr1*-*sgr9* represent a series of defective shoot gravity perception mutants with abnormal branch angles^5^. The vacuolar membrane dynamics of the stem gravity-sensing cells of the *sgr2*, *sgr3*, *sgr4* and *sgr8* mutants are abnormal and affect the sedimentable movements of statoliths (amyloplasts)^5^. In the *sgr9* mutant, interaction between statoliths and actin filaments is perturbed, resulting in attenuated statolith sedimentation^28^. In the present study, two orthologues of *SGR4*, *BnaA04g09380* and *BnaC04g31610*, are located at 8.4 Mb on A04 and 33.4 Mb on C04, 205.4 kb downstream from the peak SNP Bn-A04-p6929056 and 370.9 kb upstream from the peak SNP Bn-scaff_16876_1-p1162532, respectively (Supplementary Data S7). We also identified an orthologue of *SGR2* at 13.6 Mb on C08, which is 302.7 kb downstream from the peak SNP Bn-scaff_16468_1-p450133 (Supplementary Data S7). In addition, the *SGR9* orthologue *BnaA10g26980* was identified at 17.1 Mb on A10, 132.8 kb upstream from the peak SNP Bn-A10-p17414621 (Supplementary Data S7). Notably, the loci harbouring the *SGR4* orthologue on C04 and the *SGR9* orthologue on A10 were both identified by stringent MLM. In addition, the orthologues of *Arabidopsis SGR3* and *SGR5* were detected within the LD decay of the SNPs at 18.1 Mb on A02 and 22.7 Mb on A06 by using the A-D test, respectively, though the corresponding signals were not significant (−log_10_(*p*) = 4.6 < 5.6, −log_10_(*p*) = 4.8 < 5.6).

*LAZY1* is a well-characterized gene that modulates branch (tiller) angles in plants. The *Arabidopsis* mutant *lazy1* exhibits a dramatically increased branch angle of up to 81°, as compared to the 42° branch angle of wild-type plants^7^. In the present study, *BnaA10g19550* and *BnaC03g06250*, two orthologues of *LAZY1* in rapeseed, were identified at 13.9 Mb on A10 and 3.0 Mb on C03, respectively, in close proximity (3.8 kb and 27.2 kb, respectively) to their corresponding peak SNPs Bn-A10-p13818569 and Bn-scaff_18936_1-p472353 (Supplementary Data S7, Fig. 2). The orthologues of another well-known branch (tiller) angle gene *TAC1*, were also detected within the LD decay of SNPs at 0.7 Mb on A05 and 0.6 Mb on C04 by using GLM and the A-D test, respectively, though the signals were not significant (−log_10_(*p*) = 2.8 < 4.3, −log_10_(*p*) = 4.0 < 5.6).

A recent study in *Arabidopsis* showed that mutants with defects in auxin homeostasis or auxin response genes, such as *wei8 tar2*, *tir1-1*, *afb4-2 afb5-5*, *arf10-3 arf16-2*, have altered branch angles^10^. In the present study, *BnaA07g19520*, which is orthologous to the auxin receptor *TIR1*, was identified at 15.6 Mb on A07, 74.7 kb downstream from the peak SNP Bn-A07-p13662635 (Supplementary Data S7). The orthologues of another auxin receptor *AFB5* and the auxin biosynthesis gene *TAR2*, *BnaA01g13890* and *BnaA01g14030*, were identified at 7.0 Mb on A01, 107.5 kb and 30.5 kb upstream, respectively, from the peak SNP Bn-A01-p7974551 (Supplementary Data S7). We also identified the orthologue of *ARF10*, *BnaA07g13830*, at 12.2 Mb on A07, which is 118.2 kb downstream from the peak SNP Bn-A07-p10869578 (Supplementary Data S7). In addition to the abovementioned candidate genes, we identified other candidate genes orthologous to documented *Arabidopsis* genes involved in auxin homeostasis or signalling pathways, such as *ARF10*, *AXL1*, *RUB1*, *ARF9*, *IAA13*, *IBR10*, *ILL2*, *SAUR30*, *SAUR60*, *GSL8*, *ABCB1*, *ABCB14*, *ABCB19*, *ABCB21*, *WAT1*, *PIN3*, *PIN7*, *APD7*, *HA1*, *HA2*, *CRK5*, *VAMP714*, *UGT74D1* and *UGT84B1* (Supplementary Data S7). Moreover, the loci harbouring the *VAMP714*, *SAUR30* and *UGT74D1* orthologues on A03, the *IBR10* orthologue on A04, the *ABCB14* orthologue on C05 and the *ABCB19* orthologue on C09 were identified by MLM (Supplementary Data S7, Fig. 2).

## Discussion

The ideotype theory has prompted crop geneticists to map and clone plant type-related QTLs. To date, several genes that control the rice tiller angle, including *LAZY1*^6^, *TAC1*^8^ and *PROG1*^29^, have been cloned in biparental segregating populations. Because of the limited genetic diversity between two parents, natural populations or nest association mapping (NAM) populations have been developed to identify more favourable genes for plant-type traits. Tian *et al.* performed GWAS and joint linkage mapping of leaf traits, including the leaf angle trait, in maize NAM populations derived from 25 founder lines. A total of 203 significant SNPs and 30 QTLs were identified, including the known genes *LG1* and *LG2*^15^. Huang *et al.* conducted GWAS of various traits, including the tiller angle trait, using 446 diverse *O. rufipogon* accessions. Several loci, including the well-characterized major gene *PROG1*, were detected by using the stringent compressed MLM model^13^. In the present study, we performed GWAS in 520 diverse rapeseed accessions to reveal the QTLs affecting the branch angle trait. Based on the results of GWAS and GO annotation, we identified 77 plausible genes underlying the abundant phenotypic variation, including orthologues of well-characterized *Arabidopsis* genes, such as *SGR2*, *SGR4*, *SGR9*, *LAZY1*, *TIR1*, *AFB4*, *PIN3* and *PIN7*.

The phenotypic data obtained for branches at different positions enabled us to determine the branch(es) that provide the most reliable representation of the branch angle phenotype. High correlation (r = 0.93) was observed between the average angle of five branches from the top and the average angle of all branches, and measuring only these five branches rather than all branches would reduce the time required to perform such an evaluation and preclude confounding due to the variable branch numbers of different plants. Our statistical analyses revealed that plant height and branch angle are positively correlated, suggesting that shorter accessions tend to accompany a more compact canopy architecture. In addition, we also observed positive correlations between plant-type traits and yield-related traits. These results are informative to breeders attempting to adapt branch angles to achieve the ideal canopy architecture and high yields.

The association panel examined in the present study exhibited a strong LD of up to 6,660 kb (1,046 and 7,882 kb for the A and C subgenomes) at a cut-off value of r^2^ = 0.1, which can be explained by three possible reasons. First, the often cross-pollination habit of rapeseed (natural outcrossing rate of 10–30%) could partially account for this phenomenon, as limited recombination from inadequate outcrossing is insufficient to break strong LD. The similar phenomenon has been observed in crops with low natural outcrossing rate, such as rice, which showed residual LD at a distance of 2,000 kb^21^. Second, the majority of accessions in the present association panel are Chinese elite breeding accessions; therefore, strong artificial selection for certain traits, such as punctual flowering time, high yield, and low erucic acid and glucosinolate levels, has exerted strong selective sweeps on the flanking regions of favourable genes and consequently has caused strong LD. Similar phenomena have been observed in maize, with LD decay ranging from less than 1 kb in landraces^30^ to more than 100 kb in elite breeding lines^31^. Third, the “founder effect” could also account for the strong LD in our association panel. According to Liu *et al.*, *B. napus* was first introduced to China from Japan and Europe in the 1930–1940s^32^, and 97.3% of the certified cultivars from 1953 to 1982 were derived from one Japanese cultivar Shengli (based on a report published in Chinese in 1985). In addition, to lower seed erucic acid and glucosinolate contents, several cultivars carrying the corresponding favourable alleles from Canada or Europe, including Oro, Tower, Midas, Marnoo and Reyent, were introduced to China as the main donor parents for crossing with local elite lines. A partial estimate indicated that more than 39.7% of the 63 certified low erucic acid or glucosinolate cultivars from 1985 to 1996 are directly derived from these donor parents^33^. Consequently, the “founder effect” may have caused the strong LD observed in our association panel. Furthermore, the last two explanations might also be responsible for the stronger LD observed in our association panel as compared to that of several reported *B. napus* populations^34^,^35^,^36^.

The 56 GWAS loci in the present study cumulatively explained up to 51.1% of the phenotypic variation when using a simple additive model. Interestingly, despite the numerous significant SNPs identified, none of them explained more than 7.0% of the phenotypic variation. The significant SNPs identified by GLM and MLM explained 2.6–6.6% and 3.2–6.0% of the phenotypic variation, respectively (Table 4). Similar genetic architectures have been observed for other complex traits, such as maize leaf angle, whereby 96.0% of the 30 significant QTLs had less than a 2.5° effect^15^. In addition, the 29 large branch angle lines in the association panel have favourable alleles for 32.8 ± 5.6 (±SD) of the 56 loci associated with branch angle, whereas the 27 small branch angle lines have 17.9 ± 4.9 (±SD) favourable alleles (Supplementary Fig. S2). Accordingly, these results suggest that the observed large differences in branch angle among inbred rapeseed lines are not caused by merely a few genes with large effects, but rather by the cumulative effects of numerous QTLs having only small individual impacts on the trait.

In the present study, three methods (MLM, GLM and the A-D test) were collectively applied to dissect the genetic architecture of branch angle. Considerable GWAS loci (described in the Results) overlapped between the methods; however, we also observed variations in the power and applicable scenarios of these three methods. Compared with GLM and particularly MLM, the A-D test showed two advantages. First, because the A-D test is a nonparametric test, it is more robust, particularly for traits with abnormal phenotypic distributions and controlled by moderate effects or rare variations^24^. For example, two rare genes *BnaC08g09050* and *BnaC08g44370* (MAF = 0.09, 0.08, respectively), which are orthologous to well-known *Arabidopsis SGR2*^37^ and *5PTASE13*^38^, were solely detected in the A-D test. Second, population structure has profound effects on the results of GWAS, as reported in a previous study^34^. Because the A-D test does not include a correction for population structure, *p-*value overcorrections do not occur when the method is applied to traits that are correlated with population structure^39^. Nonetheless, MLM performed better than the A-D test for major QTLs, particularly those with common alleles^24^, such as *BnaC04g31610* and *BnaC07g15160* (MAF = 0.29, 0.35, respectively). Compared with GLM, MLM is more stringent because it involves familial relationships (kinship) to further reduce *p*-value inflation, which caused false negative results in the present study. For example, the loci harbouring *BnaA07g19520*, *BnaA10g19550* and *BnaA10g26980* failed to reach the significance threshold in MLM but not in GLM, even though the orthologues of these genes in *Arabidopsis*, *TIR1*^10^, *LAZY1*^40^ and *SGR9*^28^, have well-documented roles in branch angle regulation. Because the robust A-D test and GLM may introduce more false positives, it is feasible to combine the A-D test and GLM with MLM to maximize the detection power and take notice of potential false positives.

Despite the large number of loci associated with the branch angle trait, few loci were consistently detected in all environments and a considerable number of loci were only detected in one environment (Table 4). There are three possible reasons for these results. First, because GLM and the A-D test are permissive models that potentially introduce false positives, certain loci may have represented spurious signals that reflect confounding population structure. Second, environment can influence QTL expression and its magnitude because environment represents the manifestation of complex biotic, abiotic and agronomic factors^41^. Indeed, environmental effects are evident in previous QTL mapping analyses^41^,^42^,^43^. In the present study, ANOVA analysis revealed that environment did have significant effects on branch angle; therefore, the expression of certain sensitive QTLs could be affected by environment. Third, the GWAS model chosen can also affect the results because different models are proposed based on different statistical assumptions. For example, the locus harbouring one copy of *LAZY1* on C03 was detected in two environments (2012–2013 Nanjing and 2013–2014 Wuhan) by using the A-D test but not by using GLM.

Branch angle is a special gravitropic set-point angle (GSA) representing a plant architecture trait that is primarily governed by plant gravitropism; however, the precise mechanisms underlying branch angle maintenance and development remain poorly understood. Interestingly, a recent study proposed a model to address this issue^10^, showing that an auxin-dependent antigravitropic response acts antagonistically with the gravitropic response to maintain angled growth: the branch angle value is dependent on the magnitude of the antigravitropic response and is mediated via TIR1/AFB-AUX/IAA-ARF-dependent auxin signalling pathway within stem endodermal cells^10^. Intriguingly, in the present study, the orthologues of *Arabidopsis* auxin signalling genes, including *TIR1*, *AFB4*, *AXL1*, *RUB1*, *ARF9*, *ARF10* and *IAA13*, were collectively identified as candidate genes (Supplementary Data S7). Although the precise mechanism underlying the antigravitropic response is not fully understood, this model provides a conceptual framework for understanding the mechanism responsible for the branch angle trait and highlights a new avenue for further research.

## Methods

### Plant materials

A set of 530 diverse rapeseed inbred accessions, including landraces and elite varieties, was collected to construct the association panel, a subset of which was reported in a previous study of flowering time^44^. Based on the information obtained for these varieties, plants were assigned to three germplasm types: winter type (41), semi-winter type (435) and spring type (54). The origins of the plants showed that 485 accessions originated from Asia, 32 from Europe, 8 from North America and 5 from Australia (Supplementary Data S4). Remarkably, China contributed 476 accessions that originated from three rapeseed sub-regions with diverse climates, land fertilities and hydrologies, and these accessions broadly represent the major genetic diversity of the Chinese rapeseed gene pool.

### Experimental design and trait measurement

In 2011/2012 in Wuhan, we measured the branch angles of 30 randomly selected lines at three weeks after the final flowering stage (Supplementary Data S1). A small section from the base of the stem encompassing each branching node was photographed and analysed using Photoshop to measure the adaxial angle of the branch to the stem. Correlation analysis between the angles of branches at different positions was performed in R^44^.

The association panel was grown in the 2012/2013 and 2013/2014 growing season using a randomized complete block design with three replications on experimental farms at Changsha (N 28.22°, E 113.00°), Wuhan (N 30.52°, E 114.32°) and Nanjing (N 32.05°, E 118.78°) China. Meteorological data for the three locations are presented in Supplementary Data S8. Each line was grown in a plot with two rows and 12–15 plants in each row. The phenotypic investigation started approximately three weeks after the final flowering stage. In 2012/2013, we measured the five branches from the top of each plant, and four plants for each accession from two replicates were selected. In 2013/2014, we extended the sample size to 12.

Correlation analysis and analysis of variance (ANOVA) of branch angle for the association panel across different environments was performed in R^44^. Subsequently, an R script based on a linear model was used to obtain the broad-sense heritability and best linear unbiased prediction (BLUP) of the multi-environment phenotypes for each accession^45^. The BLUPs and individual environment data were used as the phenotypes for association analysis. Pearson’s correlation coefficient between branch angle and multiple agronomic traits, including plant height, branch number, main inflorescence pod number, pod length, seed number per pod, seed weight and seed yield (only data from 2012/2013 and 2013/2014 at Changsha were available for these traits), were calculated in R^44^.

### SNP genotyping, filtering and in silico mapping

Leaf tissue samples from the entire association panel were obtained from bulk of at least four individuals for each accession at the seedling stage. DNA was extracted using a modified CTAB procedure according to Murray and Thompson^46^. The DNA quality was carefully assessed prior to genotyping.

SNP genotyping was performed using the Illumina *Brassica* 60K Infinium^®^ SNP array according to the manufacturer’s instructions (http://www.illumina.com/technology/infinium_hd_ assay.ilmn). SNP data were clustered and automatically called using Illumina Genome Studio genotyping software. First, accessions with a Call Rate < 0.7 were excluded and all SNPs were reclustered. Next, SNPs with Call Freq < 0.75, Minor Freq < 0.05, AA or BB frequencies < 0.03 or GenTrain Scores < 0.5 were excluded. The remaining SNPs were manually reassessed, and those that did not show three clearly defined clusters were also excluded. Because heterozygous SNPs cannot be distinguished from hemi-SNPs or false calls, heterozygous calls were treated as missing values.

Fifty base pair sequences of retained SNPs after filtration were used to perform a BLASTN^47^ search against the *B.napus* genome database (http://www.genoscope.cns.fr/brassicanapus/). Using an *e*-value threshold of *e*^−12^, SNPs corresponding to multiple loci in the genome were excluded, and only the top blast hits were retained for further analysis.

### Population structure, kinship and LD decay

The filtered SNP dataset for selected accessions with a Call Rate > 0.7 was entered into STRUCTURE V2.3.3^48^. Five independent runs were performed with a K-value (the putative number of genetic groups) varying from 1 to 10, with the length of the burn-in period and the number of MCMC (Markov Chain Monte Carlo) replications after burn-in both to 100,000 iterations under the admixture model. The most likely K-value was determined using the log probability of data [LnP(D)], and the *ad hoc* statistic Delta K, which is based on the rate of change of LnP(D) between successive K-values. The cluster membership coefficient matrices of five independent runs from STRUCTURE were integrated to obtain a Q matrix by the CLUMPP software^49^. The proportion of phenotypic variation that contributed by population structure was calculated via the lm function in R^44^. Relative kinship coefficients (K) were calculated using the SPADeGi software package^50^. All negative values between individuals were set to 0. The linkage disequilibrium measurement parameter r^2^ was used to estimate linkage disequilibrium (LD) of A and C subgenome chromosomes via TASSEL5.0^51^. When calculating LD for a specific chromosome, the LD window size was adjusted to the chromosomal SNP number to force the calculation for all marker pairs. Locally paired scatterplot smoothing in R was employed to obtain a graphical representation of LD curves.

### Genome-wide association study

Trait-SNP association analysis was performed using three methods. Both GLM and MLM were implemented in TASSEL 5.0^51^. GLM takes into account population structure as a fixed effect. On this basis, MLM incorporates kinship as a random effect to further eliminate the excess of low *p*-values^19^. For GLM and MLM, the significance of associations between SNPs and traits was based on the uniform threshold *p* ≤ 5.2 × 10^−5^ (−log_10_(*p)* = 4.3). The A-D test for branch angle was conducted by using the R package ADGWAS 1.0^24^. The A-D test is a nonparametric test that includes no correction for population structure. A more stringent threshold was set for the A-D test with *p* ≤ 2.6 × 10^−5^ (−log_10_(*p)* = 5.6).

To better understand the explanatory power of the significant SNPs, we used the SNP genotypes at candidate loci as predictor variables in multiple linear models fitted to the phenotypic variables and subsequently ran a model comparison analysis (stepwise AIC procedure implemented as the R function “stepAIC”)^52^ to determine the best fitting model. The adjusted R^2^ of the best fitting multiple regression model was referred to as the phenotypic variation explained by the significant SNPs.

### Candidate gene mining

To define regions of interest containing potential candidate genes, local LD decay was calculated within flanking regions up to 12,000 kb on either side of significant SNPs using TASSEL5.0^51^, and a cut-off value of 0.1 was used for the LD statistic r^2^. Genes within the observed LD decay were annotated using the software Blast2GO v3.3.5 with the default settings^53^. In particular, genes with GO terms for gravitropism, amyloplast, and auxin were highlighted. Using the whole genome genes as reference, GO enrichment analysis of all genes within the LD decay ranges was implemented using Fisher’s Exact Test in Blast2GO v3.3.5 (false discovery rate < 0.05)^53^. Next, we performed BLASTX searches against the *Arabidopsis* genome to determine whether candidate SNP-tagged genome regions contain genes orthologous to *Arabidopsis* genes with established roles in shoot gravitropism. Additionally, we exploited the *Arabidopsis* “Electronic Fluorescent Pictograph” (eFP) browser^54^ and microarray data from previously published gravistimulation studies^55^,^56^,^57^ to further characterize candidate genes. Notably, associated SNPs that are not in or near branch angle-related genes within the LD decay (r^2^ = 0.1) were considered linked to a more distant gene, and the closest one of these genes was considered the most likely candidate.

## Additional Information

**How to cite this article**: Sun, C. *et al.* Genome-Wide Association Study Dissecting the Genetic Architecture Underlying the Branch Angle Trait in Rapeseed (*Brassica napus* L.). *Sci. Rep.* **6**, 33673; doi: 10.1038/srep33673 (2016).

## Supplementary Material

Supplementary Information

Supplementary Dataset S1

Supplementary Dataset S2

Supplementary Dataset S3

Supplementary Dataset S4

Supplementary Dataset S5

Supplementary Dataset S6

Supplementary Dataset S7

Supplementary Dataset S8

Supplementary Dataset S9

Supplementary Dataset S10

## Figures and Tables

**Table 1:** Correlations between the angles of different branches.

Trait^a^	ALLBA	1-5BA	1-4BA	1-3BA	1-2BA	1BA	2BA	3BA	4BA
1-5BA	0.93***								
1-4BA	0.92***	0.99***							
1-3BA	0.86***	0.95***	0.98***						
1-2BA	0.79***	0.90***	0.94***	0.97***					
1BA	0.71***	0.81***	0.84***	0.86***	0.92***				
2BA	0.75***	0.85***	0.88***	0.92***	0.92***	0.69***			
3BA	0.81***	0.85***	0.86***	0.85***	0.69***	0.54**	0.72***		
4BA	0.90***	0.89***	0.86***	0.74***	0.68***	0.64***	0.61***	0.71***	
5BA	0.78***	0.83***	0.72***	0.64***	0.57***	0.51**	0.53***	0.65***	0.79***

^a^ALLBA: average angle of all branches; 1-5BA: average angle of the five branches from the top; 1-4BA: average angle of the four branches from the top; 1-3BA: average angle of the three branches from the top; 1-2BA: average angle of the two branches from the top; 1BA: angle of the first branch from the top; 2BA: angle of the second branch from the top; 3BA: angle of the third branch from the top; 4BA: angle of the fourth branch from the top; 5BA: angle of the fifth branch from the top. The significance level: *p ≤ 0.05; **p ≤ 0.01; ***p ≤ 0.001.

**Table 2:** Phenotypic variations in branch angle for the rapeseed panel in four environments.

Environment	Min ± SD (°)	Max ± SD (°)	Mean ± SD (°)	CV (%)
2012/2013 Changsha	28.4 ± 2.6	71.7 ± 4.8	43.2 ± 6.3	14.5
2012/2013 Nanjing	21.7 ± 1.9	59.6 ± 3.8	40.7 ± 6.6	16.2
2013/2014 Changsha	26.0 ± 3.2	57.7 ± 6.0	40.3 ± 6.3	15.7
2013/2014 Wuhan	27.2 ± 3.3	56.8 ± 3.1	41.6 ± 6.3	15.1

**Table 3:** Correlations between branch angle and other traits in the 2012/2013 Changsha samples.

Trait	BA	PH	BN	MIPN	PL	SNPP	SW
PH	0.25***						
BN	0.17***	0.26***					
MIPN	0.11***	0.29***	0.02				
PL	0.11***	0.19***	0.01	−0.03			
SNPP	0.13***	0.23***	0.04	−0.02	0.60***		
SW	0.04	0.00	−0.14***	0.00	0.26***	0.13***	
SY	0.10**	0.37***	0.27***	0.29***	0.29***	0.29***	0.17***

BA: branch angle; PH: plant height; BN: branch number; MIPN: main inflorescence pod number; PL: pod length; SNPP: seed number per pod; SW: seed weight; SY: seed yield. The significance level: *p ≤ 0.05; **p ≤ 0.01; ***p ≤ 0.001.

**Table 4:** SNPs significantly associated with branch angle.

QTLs	SNPs	Chr	Position	MAF^a^	Allele	−log10(*p*) (MLM)	Environment (MLM)^b^	R^2^ (MLM)	−log10(*p*) (GLM)	Environment (GLM)^b^	R^2^ (GLM)	−log10(*p*) (A-D test)	Environment (A-D test)^b^
1	Bn-A01-p7430311	A01	6794653	0.32	[A/G]				5.1–5.7	BLUP,CS14	3.9–5	6.1–6.4	BLUP,CS14
2	**Bn-A01-p7974551**	A01	7156865	0.42	[T/C]				4.3–4.4	CS14,WH14	3.1–3.7	6.0–7.1	BLUP,NJ13,WH14
3	**Bn-A02-p27245861**	A02	24567443	0.30	[A/G]							5.7–7	BLUP,NJ13,WH14
4	Bn-A03-p4342338	A03	3874153	0.32	[T/C]	5.5	BLUP	4.9	5.0–6.2	BLUP,NJ13	3.7–5.1		
5	Bn-A03-p5814447	A03	5245728	0.41	[A/G]				4.3	BLUP	3.3		
6	**Bn-A03-p6228570**	A03	5594660	0.27	[T/C]	4.7–6.3	BLUP,CS13	3.6–3.9	4.4–5.5	BLUP,CS13,NJ13	2.9–3.3	5.6–5.7	BLUP,NJ13
7	Bn-A03-p7178650	A03	6451720	0.16	[T/C]				4.7–5.2	BLUP,NJ13	3.4–4.0		
8	**Bn-A03-p7572128**	A03	6882418	0.10	[T/G]	4.3	BLUP	3.2	5.5–6.3	BLUP,NJ13,WH14	3.5–4.0		
9	**Bn-A03-p8033095**	A03	7321907	0.27	[T/C]				5.0–5.1	BLUP,NJ13	3.7–3.9	6.2–6.8	BLUP,NJ13,WH14
10	**Bn-A03-p8554739**	A03	7848675	0.33	[T/C]				4.5–6.0	BLUP,CS13,WH14	3.6–4.6	6.5–7.1	BLUP,WH14
11	Bn-A03-p15347967	A03	14385059	0.30	[A/G]							6.2	BLUP
12	Bn-A03-p25868537	A03	24234777	0.27	[A/G]				4.5–5.7	BLUP,WH14	3.4–4.3		
13	**Bn-A03-p28982546**	A03	27392934	0.24	[T/C]				5.4–5.7	BLUP,CS13,NJ13	4.0–4.5		
14	**Bn-A04-p3631563**	A04	3299951	0.13	[A/G]	4.8–6.7	BLUP,NJ13,CS13	4.1–6.0	4.5–8.6	BLUP,CS13,NJ13,CS14	3.5–6.6		
15	Bn-A04-p4410144	A04	4532791	0.27	[T/C]	4.7–5.5	BLUP,NJ13	4.2–4.9	6.0–6.4	BLUP,NJ13	4.6–5.2	5.9	NJ13
16	**Bn-A04-p2411039**	A04	5850169	0.14	[A/G]	4.6–4.8	BLUP,NJ13,CS13	3.8–4.3	5.5–6.3	BLUP,CS13,NJ13	4.3–4.9		
17	**Bn-A04-p6296526**	A04	7519507	0.23	[A/C]	4.9	BLUP	3.7	4.8–4.8	CS13,NJ13	3.2–3.2		
18	Bn-A04-p6929056	A04	8211241	0.35	[T/C]				4.4	BLUP	3.5		
19	Bn-A05-p6415983	A05	5963980	0.07	[A/G]				4.6	BLUP	3.0		
20	Bn-A05-p21065087	A05	19223021	0.43	[A/G]				4.6	BLUP	3.7		
21	Bn-A06-p5114770	A06	4927748	0.27	[T/C]				4.4	BLUP	3.4		
22	Bn-A10-p11839950	A07	2293313	0.27	[T/C]				4.5–4.5	BLUP,WH14	3.5–3.6		
23	**Bn-A07-p4191541**	A07	6111458	0.47	[T/C]				5.7–7.6	BLUP,NJ13,WH14	4.7–6.4	6.7–7.7	BLUP,NJ13,WH14
24	**Bn-A07-p5412930**	A07	7273126	0.47	[A/G]				4.6–5.1	BLUP,CS13,WH14	3.4–4.1	5.8–6.6	BLUP,CS13,WH14
25	Bn-A07-p9921856	A07	11205489	0.24	[T/G]				4.4–5.6	BLUP,NJ13	2.7–3.8		
26	Bn-A07-p10869578	A07	12103546	0.27	[T/C]				4.5–4.7	BLUP,WH14	3.3–3.6		
27	**Bn-A07-p13172047**	A07	15255974	0.22	[A/G]	5.1	CS13	4.5	4.9–5.7	BLUP,CS13,WH14	3.6–4.5	6.1	CS13
28	Bn-A07-p13662635	A07	15655194	0.17	[T/C]				5.4–5.5	BLUP,WH14	3.9–3.9		
29	Bn-A07-p15007983	A07	16890653	0.32	[T/G]							5.6	BLUP
30	Bn-A07-p15505090	A07	17408302	0.46	[T/G]				4.3	BLUP	2.6		
31	Bn-A07-p18021746	A07	19874380	0.33	[T/C]				4.7–5.1	BLUP,CS14	3.7–4.5		
32	Bn-A08-p13638847	A08	11390087	0.29	[T/G]				4.3–6.7	BLUP,CS14	2.6–4.7	5.5	BLUP
33	Bn-A09-p2208929	A09	1382771	0.20	[A/G]				4.4–5.2	BLUP,CS14	2.9–3.9		
34	**Bn-A09-p36803825**	A09	33753723	0.09	[T/C]	4.9–5.4	BLUP,CS14,CS13	3.9–4.3	4.5–7.7	BLUP,CS13,NJ13, CS14,WH14	3.2–5.1		
35	Bn-A10-p7394578	A10	9067281	0.48	[A/G]				4.3–5.2	BLUP,WH14	3.3–4.1		
36	Bn-A10-p10671726	A10	12042143	0.15	[T/C]				4.4–6.6	BLUP,CS13	3.2–5.3		
37	Bn-A10-p13818569	A10	13855272	0.40	[T/C]				4.6–4.6	BLUP,CS13	4.0	5.6–6.3	BLUP,CS13
38	Bn-A10-p17414621	A10	17217158	0.08	[T/C]	4.4	NJ13	3.6	4.8–6.3	BLUP,NJ13	3.3–4.6		
39	Bn-scaff_20461_1-p322463	C02	9345306	0.11	[A/C]				5.6	BLUP	3.5		
40	Bn-scaff_16614_1-p1480092	C03	660277	0.08	[T/G]				4.5–5.1	BLUP,WH14	2.7–3.2		
41	**Bn-scaff_18936_1-p472353**	C03	3058478	0.32	[A/G]				4.6	BLUP	3.3	6.2–8.7	BLUP,NJ13,WH14
42	Bn-scaff_18322_1-p2487448	C03	6744282	0.40	[A/C]							6.2–6.6	BLUP,WH14
43	Bn-scaff_15794_3-p533873	C03	55055879	0.34	[T/C]				4.4	BLUP	3.2		
44	Bn-scaff_16876_1-p1162532	C04	33772508	0.29	[A/C]	4.4–5.8	BLUP,CS13	3.3–4.5	6.3	CS13	4.2		
45	**Bn-scaff_15798_1-p281492**	C04	36910519	0.15	[T/C]	5.0–5.0	BLUP,CS14	4.3–4.3	5.7–7.5	BLUP,CS14,WH14	4.4–5.3		
46	**Bn-scaff_16062_1-p345501**	C05	15699504	0.40	[A/G]	4.9	CS13	4.5	4.4–7.4	BLUP,CS13,CS14,WH14	3.2–6.2	6.1–7.3	BLUP,CS13
47	Bn-scaff_18206_1-p453711	C06	18723815	0.45	[T/C]							6.0–6.3	BLUP,WH14
48	Bn-scaff_19106_1-p76331	C07	18982139	0.26	[A/G]							6.3–9.9	BLUP,NJ13
49	Bn-scaff_18501_1-p114150	C07	21145676	0.35	[A/G]	4.6–5.7	BLUP,NJ13	3.9–5.0	4.6–5.3	BLUP,NJ13	3.4–4.2		
50	Bn-scaff_16468_1-p450133	C08	13342433	0.09	[T/G]							5.6	BLUP
51	**Bn-scaff_16200_1-p208618**	C08	27277109	0.19	[T/C]				4.3–5.2	BLUP,CS13,NJ13,CS14	3.4–4.2	5.6	NJ13
52	**Bn-scaff_16197_1-p2492352**	C08	31718611	0.47	[T/C]	4.5	BLUP	3.9	4.3–5.8	BLUP,CS13,CS14	3.2–4.7		
53	**Bn-scaff_16197_1-p1333425**	C08	32784282	0.28	[A/T]				4.6–5.3	BLUP,NJ13,WH14	3.4–3.9	6.1	WH14
54	Bn-scaff_16389_1-p436578	C08	37667324	0.08	[A/G]							5.9–6.1	BLUP,WH14
55	**Bn-scaff_17526_1-p1470085**	C09	1151339	0.35	[T/G]	5.0	WH14	3.8	5.7–8.0	BLUP,CS14,WH14	3.9–5.2		
56	Bn-scaff_16362_1-p621813	C09	43501182	0.20	[A/G]				5.4	WH14	3.8	5.6–6.0	BLUP,WH14

^a^MAF: minor allele frequency.

^b^NJ13: 2012/2013 Nanjing, CS13: 2012/2013 Changsha, CS14: 2013/2014 Changsha, WH14: 2013/2014 Wuhan. SNPs indicated in bold were consistently detected in at least two environments in the present study.
