# Supplementary material for: Genome-Wide Association Study Dissecting the Genetic Architecture Underlying the Branch Angle Trait in Rapeseed (Brassica napus L.)
Source: Sci Rep. 2016 Sep 20;6:33673. doi: 10.1038/srep33673 (PMC5028734; doi:10.1038/srep33673)
Supplement: Supplementary Information [file srep33673-s1.pdf]

Supplementary Information for

Genome-Wide Association Study Dissecting the Genetic Architecture Underlying the Branch Angle Trait in Rapeseed (*Brassica napus* L.)

Chengming Sun[1], Benqi Wang[1], Xiaohua Wang[1], Kaining Hu[1], Kaidi Li[1], Zhanyu Li[1], San Li[1], Lei Yan[1], Chunyun Guan[2], Jiefu Zhang[3], Zhenqian Zhang[2], Song Chen[3], Jing Wen[1], Jinxing Tu[1], Jinxiong Shen[1], Tingdong Fu[1] and Bin Yi[1,*]

[1]Huazhong Agricultural University, National Key Laboratory of Crop Genetic Improvement, National Sub-center of Rapeseed Improvement in Wuhan, Wuhan, 430070, China

[2]Hunan Agricultural University, College of Agronomy, Changsha, 410128, China

[3]Jiangsu Academy of Agricultural Science, Key Laboratory of Cotton and Rapeseed, Nanjing, 210014, China

*yibin@mail.hzau.edu.cn

Supplementary Figure

Supplementary Figure S1. Overall genome-wide decay of linkage disequilibrium (LD).

Supplementary Figure S2. Enhancive allele number of large and small branch angle lines.

Supplementary Figure S3. Gene Ontology (GO) enrichment diagram of genes locating within the linkage disequilibrium (LD) decay ranges of significant loci.

Supplementary Table

Supplementary Table S1. Correlations analysis of branch angle in four environments.

Supplementary Table S2. ANOVA analysis of branch angle in four environments.

Supplementary Table S3. Correlations between branch angle and other traits of 2013/2014 Changsha.

Supplementary Table S4. Linkage disequilibrium (LD) decay on A and C subgenome chromosomes in the 520 rapeseed accessions.

Supplementary Dataset

Supplementary Data S1. Branch angle of all branches for 30 lines.

Supplementary Data S2. Supplementary Data S2. The phenotypic data for all accessions in four environments and BLUP values and the broad-sense heritability for four environments.

Supplementary Data S3. Genotyping scores of all the polymorphic SNPs for the association panel.

Supplementary Data S4. Information of the 530 association panel and group information based on population structure.

Supplementary Data S5. Data of population structure and kinship for the association panel.

Supplementary Data S6. Gene Ontology (GO) enrichment analysis of genes locating within the linkage disequilibrium (LD) decay ranges of significant loci.

Supplementary Data S7. SNPs and candidate genes significantly associated with branch angle.

Supplementary Data S8. 2012/2013 and 2013/2014 meteorological data of four environments.

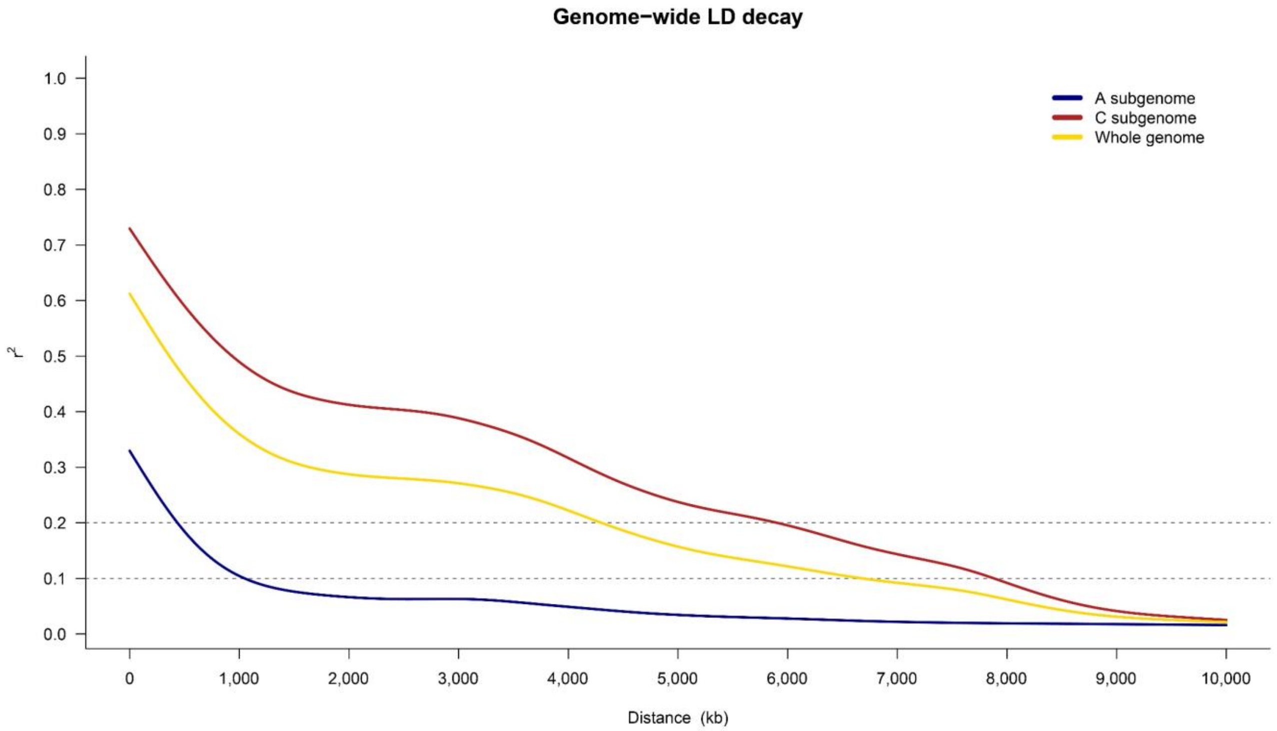

Supplementary Figure S1. Overall genome-wide decay of linkage disequilibrium (LD), shown as smoothed r^2 for all marker pairs on A, C subgenome and whole genome depending on the distance between marker pairs.

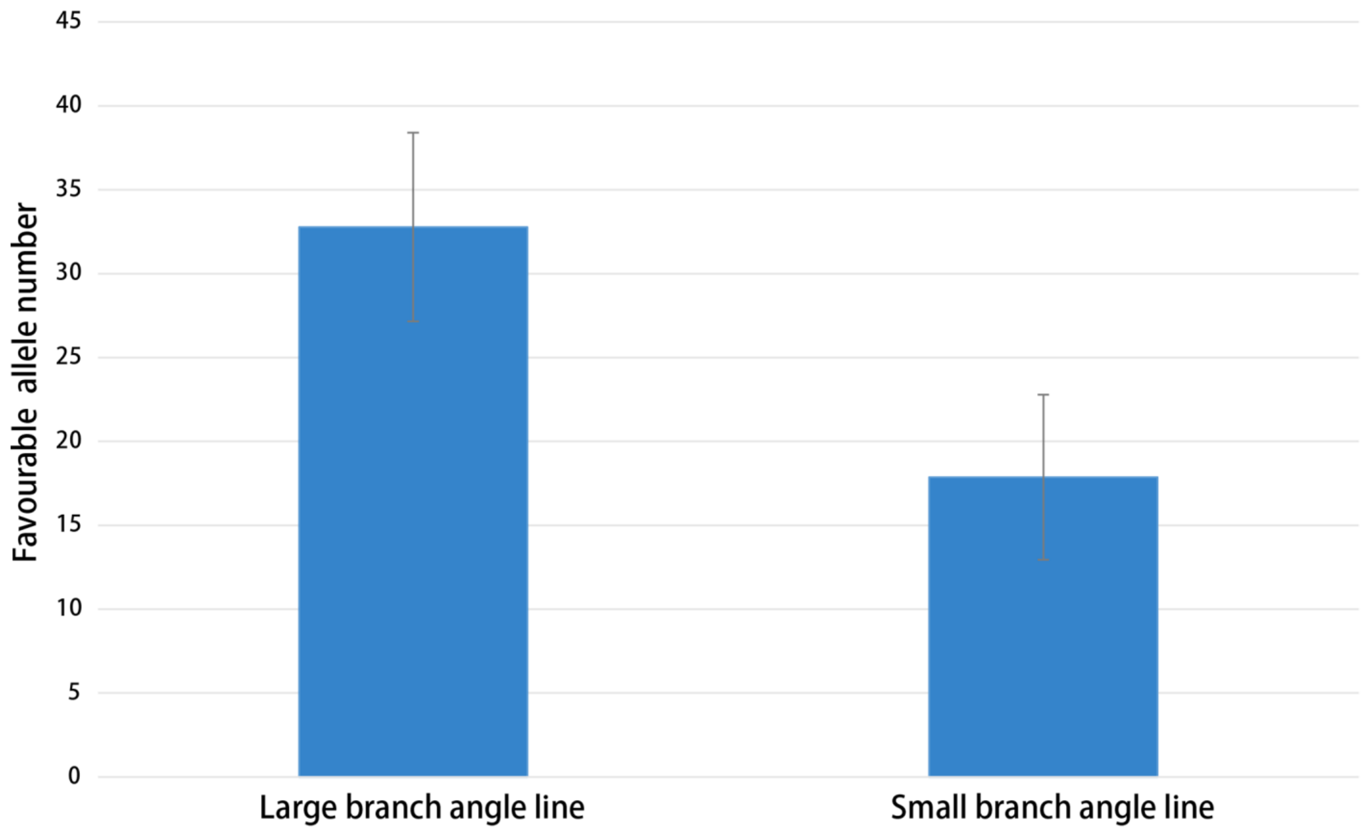

Supplementary Figure S2. Favourable allele number of large and small branch angle lines.

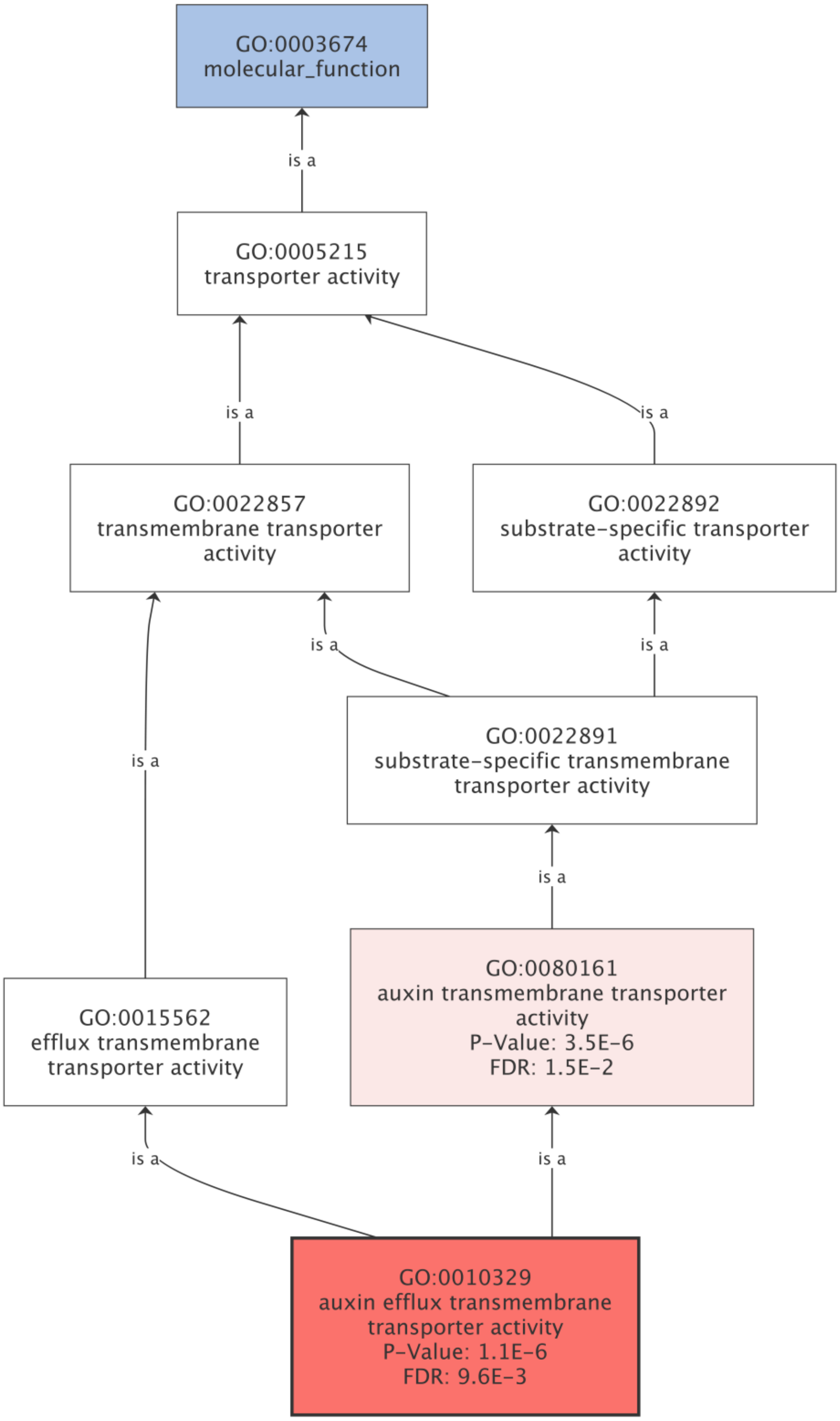

Supplementary Figure S3. Gene Ontology (GO) enrichment diagram of genes locating within the linkage disequilibrium (LD) decay ranges of significant loci.

Environment	2012/2013 Changsha	2012/2013 Nanjing	2013/2014 Changsha
2012/2013 Nanjing	0.58***		
2013/2014 Changsha	0.59***	0.54***	
2013/2014 Wuhan	0.68***	0.58***	0.63***

Supplementary Table S1. Correlations analysis of branch angle in four environments. *$p \leq 0.05$; ** $p \leq 0.01$; *** $p \leq 0.001$.

Source	DF	Sum Sq	Mean Sq	F value	Pr(>F)	
Line	519	294304	567	32.341	<2e-16	***
Loc	2	1647	824	46.968	<2e-16	***
Year	1	13117	13117	748.091	<2e-16	***
Line:Loc	1033	78106	76	4.312	<2e-16	***
Line:Year	517	30312	59	3.344	<2e-16	***
Residuals	14072	246732	18			

Supplementary Table S2. ANOVA analysis of branch angle in four environments. *$p \leq 0.05$; ** $p \leq 0.01$; *** $p \leq 0.001$.

Trait	BA	PH	BN	MIPN	PL	SNPP
PH	0.24***					
BN	0.1***	0.25***				
MIPN	0.08**	0.32***	0.08*			
PL	0.11***	0.38***	0.09**	0.09**		
SNPP	0.13***	0.32***	0.02	0.06*	0.57***	
SW	0.11***	0.18***	-0.09**	0.2***	0.31***	0.03

Supplementary Table S3. Correlations between branch angle and other traits in the 2013/2014 Changsha samples. BA: branch angle; PH: plant height; BN: branch number; MIPN: main inflorescence pod number; PL: pod length; SNPP: seed number per pod; Seed yield were not investigated. The significance level: *$p \le 0.05$; ** $p \le 0.01$; *** $p \le 0.001$.

Subgenome	Chromosome	No. of SNPs	LD decay (Kb)	
			r^2=0.1	r^2=0.2
A subgenome	A1	729	827	303
	A2	552	824	285
	A3	1041	729	225
	A4	762	827	278
	A5	761	873	356
	A6	747	796	236
	A7	939	708	254
	A8	504	4940	1254
	A9	748	2155	873
	A10	825	4264	764
	A1-A10	7608	1046	439
C subgenome	C1	1726	8278	7768
	C2	1611	8704	4493
	C3	1901	1039	507
	C4	2252	5845	4866
	C5	558	1282	732
	C6	837	1218	658
	C7	1110	6496	908
	C8	999	6944	6342
	C9	565	2968	1085
	C1-C9	11559	8278	7768
Whole genome	A1-C9	19167	6660	4311

Supplementary Table S4. Linkage disequilibrium (LD) decay on A and C subgenome chromosomes in the 520 rapeseed accessions.
